# The role of reciprocal fusions in *MLL*-r acute leukemia: studying the chromosomal translocation t(6;11)

**DOI:** 10.1038/s41388-021-01983-3

**Published:** 2021-08-05

**Authors:** Arpita Kundu, Eric Kowarz, Rolf Marschalek

**Affiliations:** Institute of Pharmaceutical Biology/DCAL, Goethe-University of Frankfurt, Biocenter, Max-von-Laue-Str. 9, D-60438 Frankfurt/Main, Germany

**Keywords:** Cancer, Cell biology

## Abstract

Leukemia patients bearing t(6;11)(q27;q23) translocations can be divided in two subgroups: those with breakpoints in the major breakpoint cluster region of *MLL* (introns 9–10; associated mainly with AML M1/4/5), and others with breakpoints in the minor breakpoint cluster region (introns 21–23), associated with T-ALL. We cloned all four of the resulting fusion genes (*MLL-AF6*, *AF6-MLL*, *exMLL-AF6*, *AF6-shMLL*) and subsequently transfected them to generate stable cell culture models. Their molecular function was tested by inducing gene expression for 48 h in a Doxycycline-dependent fashion. Here, we present our results upon differential gene expression (DGE) that were obtained by the “Massive Analyses of cDNA Ends” (MACE-Seq) technology, an established 3′-end based RNA-Seq method. Our results indicate that the PHD/BD domain, present in the AF6-MLL and the exMLL-AF6 fusion protein, is responsible for chromatin activation in a genome-wide fashion. This led to strong deregulation of transcriptional processes involving protein-coding genes, pseudogenes, non-annotated genes, and RNA genes, e.g., LincRNAs and microRNAs, respectively. While cooperation between the MLL-AF6 and AF6-MLL fusion proteins appears to be required for the above-mentioned effects, exMLL-AF6 is able to cause similar effects on its own. The exMLL-AF6/AF6-shMLL co-expressing cell line displayed the induction of a myeloid-specific and a T-cell specific gene signature, which may explain the T-ALL disease phenotype observed in patients with such breakpoints. This again demonstrated that MLL fusion proteins are instructive and allow to study their pathomolecular mechanisms.

## Introduction

T(6;11) leukemia is caused by an illegitimate recombination event between the *MLL/KMT2A* gene (11q23) with the *AF6/MLLT4/AFDN* gene (6q27). The *AF6* gene encodes the multi-domain protein Afadin that resembles a scaffold protein for connecting the actin cytoskeleton to Nectin receptors in order to build intercellular junctions (adherent junctions), similar to Cadherins with a/ß-Catenins [1–3]. The difference is found in the downstream signaling because Nectin/Afadin causes the activation of CDC42, RAC, and RAP1 signaling, while Cadherin/Catenin causes RAC/PI3K signaling [4–6].

Afadin as MLL fusion partner most likely has a different biological function, since the MLL-AF6 fusion proteins translocate into the nucleus. Of note, the nuclear translocation of MLL-AF6 also causes the arbitrary translocation of wildtype Afadin into the nucleus [7]. MLL-AF6 fusion protein was shown to interact with LIM domain proteins (e.g., LMO2) and to trigger the RAS signaling pathway, through an unknown mechanism [8]. Other groups have already described mutant *RAS* genes in leukemia patients diagnosed with t(6;11) rearrangements [9]. In addition, the AF6 fusion portion is thought to enhance the dimerization of MLL-AF6 [10, 11].

Leukemia patients bearing a t(6;11) leukemia usually display a typical AML disease phenotype with very poor prognosis (OS~10%) [12]. They display a narrow breakpoint distribution which mostly occur in *MLL* intron 9. Interestingly, 25% of all t(6;11) patients display a T-ALL disease phenotype with breakpoints scattering within *MLL*, including in the recently identified minor breakpoint cluster region (*MLL* intron 21–23) [13]. Thus, two different sets of fusion proteins can be attributed to these patient groups: the conventional MLL-AF6 and AF6-MLL fusions (breakpoint with *MLL* intron 9 or exMLL-AF6 and AF6-shMLL (breakpoints within *MLL* intron 21–23. We decided to investigate these four t(6;11) fusion proteins—alone and in combination—to learn more about their pathological role in disease onset.

In principle, these fusion proteins exhibit specific domains that define their functions. MLL-AF6 contains the MEN1/LEDGF binding domain at the very N-terminus which facilitates interaction with transcription factors bound to target gene promotors [14–16]. It also contains the CXXC domain which allows recognition and binding of hemi-methylated DNA [17–22]. The reciprocal AF6-MLL fusion protein encodes the PHD/BD domain (chromatin reading; [23–27]), with binding sites for CREBBP [28] and MOF [29] (both activating histone acetylases) as well as the SET domain [30–32]. The exMLL-AF6 contains the MEN1/LEDGF and CXXC domain in conjunction with the PHD/BD domain, while the AF6-shMLL fusion contains only the CREBBP/MOF interaction domain in conjunction with the SET domain. In summary, the difference between the two different sets of t(6;11) fusion proteins is the swapping of the PHD/BD domain from the reciprocal fusion to the direct fusion protein.

We aimed to establish an experimental model system to investigate the molecular consequences of t(6;11) fusion protein expression. The MLL wildtype protein complex is known to confer active chromatin marks on target gene promotors which enables target gene transcription [14, 33, 34]. This basic biological process is crucial for any living cell, and therefore, pathological functions deriving from t(6;11) fusion proteins should be easily monitored when investigating changes in gene transcription. This is important to mention as we did not aim to mimic leukemia development, rather to study the immediate changes on chromatin and gene transcription upon induction of fusion protein expression for only 48 h. In addition to these very basic scientific interests, we also wanted to find a rational explanation for the AML versus T-cell phenotype that were observed in diagnosed t(6;11) leukemia patients.

## Results

### Cloning and establishment of t(6;11) cell culture model systems

**Fig. 1:**
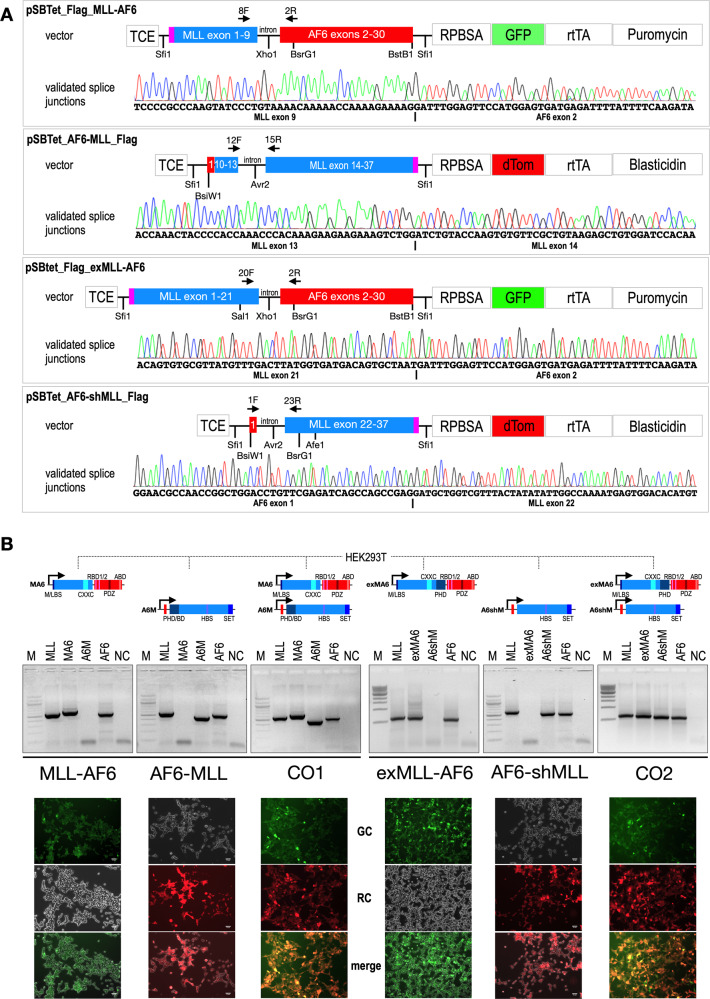
Transfected fusions genes and their expression to outline the experimental setting. **A**. All four vector constructs are depicted. Shown is only the expressed part of the different Sleeping Beauty vectors that have been used. Importantly, all inducible direct fusions were cloned into vectors that express constitutively eGFP and the Puromycin resistance genes, while the reciprocal vectors express constitutively dTom together with the Blasticidin resistance gene. Since the construction of these vectors was done by separating the two cDNA fragments by a short intronic sequence, proper splicing was validated by RT-PCR and sanger sequencing of the resulting PCR product (depicted as “validated splice junction)”. The red box with the annotation “1” represents *AF6* exon 1. Pink boxes at the beginning or end of the fusion genes indicate the presence of Flag-Tags in all constructs. **B** The construction of all 6 cell lines is shown. The encoded MLL protein domains and portions are displayed in different blue colors, while encoded Afadin/AF6 protein domains and portions are displayed in different red colors. Important MLL domains are: M/LBS: MEN1/LEDGF Binding Site; CXXC: CXXC domain; PHD/BD: PHD/BD domain; HBS: HAT Binding Site (CREBBP/MOF1); SET: SET domain). Similarly, important Afadin/AF6 domains are: RBD1/2: RAS/RAP Binding Domain 1 and 2; PDZ: PDF domain; ABD: Actin Binding domain. Besides these 6 cell lines, a control cell line with a Sleeping Beauty mock vector was established as well (pSBtet-GP). This control cell line expresses a luciferase gene instead of a fusion gene, and constitutively expresses GFP (G) together with a Puromycin resistance gene (P). Induction with 1 µg/ml Doxycyclin was carried out for 48 h in all seven cell lines before RNA or DNA was harvested to investigate changes in gene expression (MACE) or chromatin accessibility (ATAC-Seq). RT-PCR experiments were performed to validate the correct expression of all fusion transgenes next to their wildtype counterparts (endogenous genes *MLL* and *AF6*). It was important to demonstrate that all transgenes were not very highly overexpressed, rather were expressed in all cells at physiological levels. Below: Expression of the vector backbone fluorescent proteins (FP’s). Pictures were taken from all six cell lines by making photographs from the green and red channels or were merged to demonstrate the expression of the FP’s from the appropriate vector backbones. A picture of the GFP-positive mock-cell line is not displayed.

All four t(6;11) chimeric genes were cloned into so-called “universal vector backbones” which were previously established in our group [35]. Briefly, the following 4 constructs were established: [1] *MLL-AF6* (*MLL* exons 1–9::*AF6* exons 2–30), [2] *AF6-MLL* (*AF6* exon 1::*MLL* exons 10–37), [3] *exMLL-AF6* (*MLL* exons 1–21::*AF6* exons 2–30) and [4] *AF6-shMLL* (*AF6* exon 1::*MLL* exons 22–37). All 4 constructs were finally introduced into Doxycyclin-inducible pSBtet expression vectors that express additionally the combination of eGFP/Puromycin or dTom/Blasticidin [36]. Since all cloned fusion genes contained a short intronic sequence, correct splicing of all fusion genes was investigated in RT-PCR experiments and subsequent sequencing analysis of the obtained PCR fragments (see “validated splice junction” of Fig. 1A).

All these vectors were stably transfected—either alone or in combination—into HEK293T cells (ATCC CRL-3216™), together with an Luciferase control vector. As shown in Fig. 1B, all six cell lines transcribe the endogenous wildtype alleles of *MLL* and *AF6*, as well as the transfected transgenes in physiological amounts. Below the RT-PCR panels, fluorescence pictures from all six cell lines were taken in green and red channels to demonstrate the correct fluorescent protein was expressed from the vector backbones (eGFP or dTom; Fig. 1B, lower panels). Total RNA isolated from all seven cell lines (3× biological replicates) was then used for MACE-Seq analyses, or to isolate chromatin for the below described ATAC-Seq experiment (3× biological replicates).

### Outline of our experimental setting and bioinformatic pipeline: data evaluation and establishment of novel tools

As summarized in Fig. S1, our experimental setting was used to perform MACE and ATAC-Seq experiments. Differential expression analysis was performed using R-Bioconductor DESeq2 library. Raw counts were normalized by Geometric mean based method. [37]. These data were used to define a simple algorithm (more than 10 reads, *p* values < 0.05 and a log 2 fold change of ±2 that allows the definition of highly significant gene signatures. The resulting data were used to prepare Circos plots [38] for the visualization of genome-wide changes in gene transcription, or for the visualization of the ATAC-Seq data. In addition, we used these data sets to generate heatmaps, volcano plots, and pathway analyses.

In addition, we used the FileMaker database program to import all the DESeq2 data for further analysis and to apply additional algorithms. This resulted in three additional analytic modules, named GUDC, DAGT and DAGE, respectively. The GUDC module analyzes the “Gene Usage on Different Chromosomes”, which could then be graphically presented as a kind of “chromosome fingerprint” for each of the tested t(6;11) fusion proteins. In principal, this module defines the total number of genes in each data set that were deriving from each chromosomes, and any deregulated gene signature is then understood as a subset of these genes deriving from the different chromosomes (in percentage terms). The result of the analysis is then displayed for each chromosome as more (positive) or less (negative) gene expression in comparison to the mathematical mean for a given chromosome. This kind of “fingerprint” helps to understand whether genes on some chromosomes are preferentially activated, or vice versa which chromosomes are less affected by the presence of a given fusion protein. The DAGT module (“Differential Analysis by Gene Type”) automatically classifies each gene entry in our signatures to one of the different gene types (pseudogenes, non-annotated genes, LINC RNAs, MIR RNAs SNO RNAs, mitochondrial genes and protein coding genes. Finally, the DAGE module ““Differential Analysis of de novo or shut-down Gene Expression”) uses the DESeq2 data to identify “de novo induced genes” or “shut-down genes” after t(6;11) transgene expression. For this purpose, we defined a novel log2_var_ discriminator (defined as “Ln(fold change)/Ln2”) because the DESeq2 provides log2 data even when mock or experimental data displayed zero reads. By using the log2_var_ discriminator, we were able to quickly identify all “de novo transcribed genes” or “shut-down genes” and included these critical gene sets in our analyses.

### Molecular functions attributed to direct and reciprocal t(6;11) fusion proteins

**Fig. 2:**
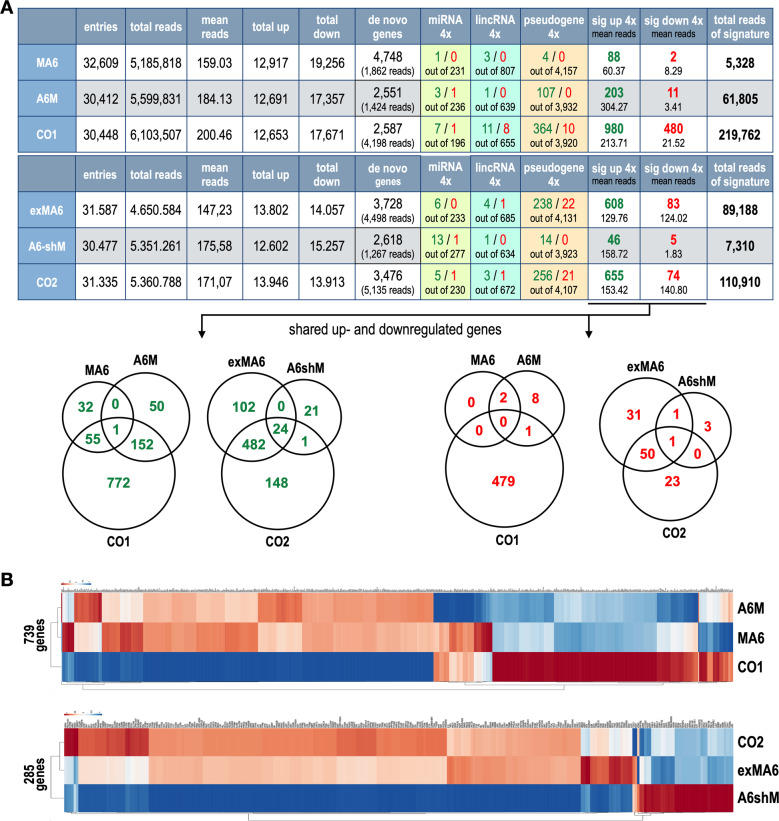
Data dissection of the MACE-Seq experiments and Heatmap analysis. **A** The obtained gene expression profile from the individual MACE experiments were summarized for comparison and data evaluation. Upper two panels: Left column: name of the transfected fusion genes or their combination (CO1 or CO2). The subsequent columns contain information about number of gene entries, total reads, mean reads, and total up- and down-regulated genes of all gene entries. The last six columns displays filtered information, as we calculated up- and down-regulated genes by a minimum of ten reads, a *p* value < 0.05 combined with a log2 changes of >2 in case of upregulated genes, while downregulated genes were identified by a minimum of 10 reads in the mock sample, a *p* value < 0.05 combined with a log2 value of <−2. We also filtered for microRNA (MIR) genes, LincRNA (LINC) genes, pseudogenes which were part of the signatures (e.g., the 88 upregulated genes in MLL-AF6 expressing cells included 1 microRNA gene, 3 LincRNA genes and 4 pseudogenes). The last columns represent the total reads of the up- and down-regulated gene signatures. Bottom panels: VENN diagrams displaying the shared up- and downregulated genes between the different signatures. A large overlap can be seen between AF6-MLL and CO1 cells, as well as with exMLL-AF6 and CO2 cells, however, only for the upregulated genes. Downregulated genes do not show such an overlap, only exMLL-AF6 and CO2 cells were found to be similar. **B** Heatmaps were created by using the gene signatures (up-and downregulated gene sets) of each individual cell line and the ClusVis online tool (biit.cs.ut.ee/clustvis/). The heatmaps were generated by using only the deregulated protein coding genes. CO1 cells differ significantly from the single transfected cells with the fusion genes MLL-AF6 and AF6-MLL, respectively. By contrast, exMLL-AF6 cells and CO2 cells cluster together and clearly separate from AF6-shMLL expressing cells. These results underscored again the synergistic (CO1) and additive effects for both sets of t(6;11) fusion protein (CO2).

The overall MACE data analysis is summarized in Fig. 2A (upper panel). It summarizes the identified number of gene entries for all 6 cell lines. The last 6 rows display the significant signatures that were identified (>10 reads, *p* value < 0.05 and FC > ±4). The analysis of the first set of t(6;11) fusion proteins clearly showed that the MLL-AF6 fusion protein generates a new and highly significant signature of 88 upregulated genes and 2 downregulated genes that comprised together 5328 reads. The reciprocal AF6-MLL fusion protein caused a signature of 203 up-regulated and 11 downregulated genes that comprised together 61,805 reads. Interestingly, the co-expression of both fusion proteins together (CO1) resulted in a gene signature with 980 up- and 480 down-regulated genes that together comprised 219,762 reads. A first conclusion from the DAGT module indicated that the reciprocal fusion protein strongly increases pseudogene (PG) usage, which is even stronger in the presence of both fusion proteins. Similarly, this was also true for the group of non-annotated genes (MLL-AF6: 15; AF6-MLL: 73; CO1: 276). Secondly, the presence of both fusion proteins resulted in a significantly larger signature and also allowed the downregulation of target genes (*n* = 480). We concluded from these results that both fusion proteins work in a synergistic fashion with each other. QRT-PCR experiments were done for a few selected target genes, solely to demonstrate the versatility of the MACE technology (Fig. S2). QRT-PCR data were highly concordant with the MACE-Seq data.

The analysis of the second set of t(6;11) fusion proteins showed quite a different situation. The expression of the exMLL-AF6 fusion alone resulted in a large signature of 608 upregulated genes and 83 downregulated genes that together comprised 88,188 reads. When the reciprocal AF6-shMLL fusion was expressed, only 46 genes were upregulated, and five genes were downregulated, comprised only 7310 reads. The co-expression of both fusion proteins (CO2) resulted again in a large signature of 655 up- and 74 down-regulated genes that comprised together 110,910 reads. A first conclusion from this analysis was that now the direct exMLL-AF6 fusion protein was responsible for the activation of large sets of pseudogenes and non-annotated genes (exMLL-AF6: 170; AF6-shMLL: 12; CO2: 165), while the reciprocal AF6-shMLL fusion protein was only able to create a minor signature of deregulated genes. Noteworthy, the reciprocal fusion protein did not work in a synergistic fashion, rather than in an additive fashion together with the direct fusion protein exMLL-AF6.

We also compared the identified gene signatures by VENN diagrams, summarized in Fig. 2A, lower panel. This type of analysis substantiated the earlier assumption.

We also used these data to create heatmaps and volcano plots. For heatmap analyses we retrieved only the protein-coding genes of all signatures. The heatmap analysis is displayed Fig. 2B, where we analyzed both sets of t(6;11) fusion proteins. The first set of t(6;11) fusions contained 739 total genes that were retrieved from the up- and down-regulated gene signatures of MLL-AF6, AF6-MLL and CO1 cells. The second set of t(6;11) fusions contained only 285 protein coding genes that were retrieved from the up- and down-regulated gene signatures of exMLL-AF6, AF6-shMLL, and CO2 cells. From these heatmap analyses it became clear that CO1 differ significantly from the single-transfected cells, while exMLL-AF6 and CO2 cells display a highly similar signature.

**Fig. 3:**
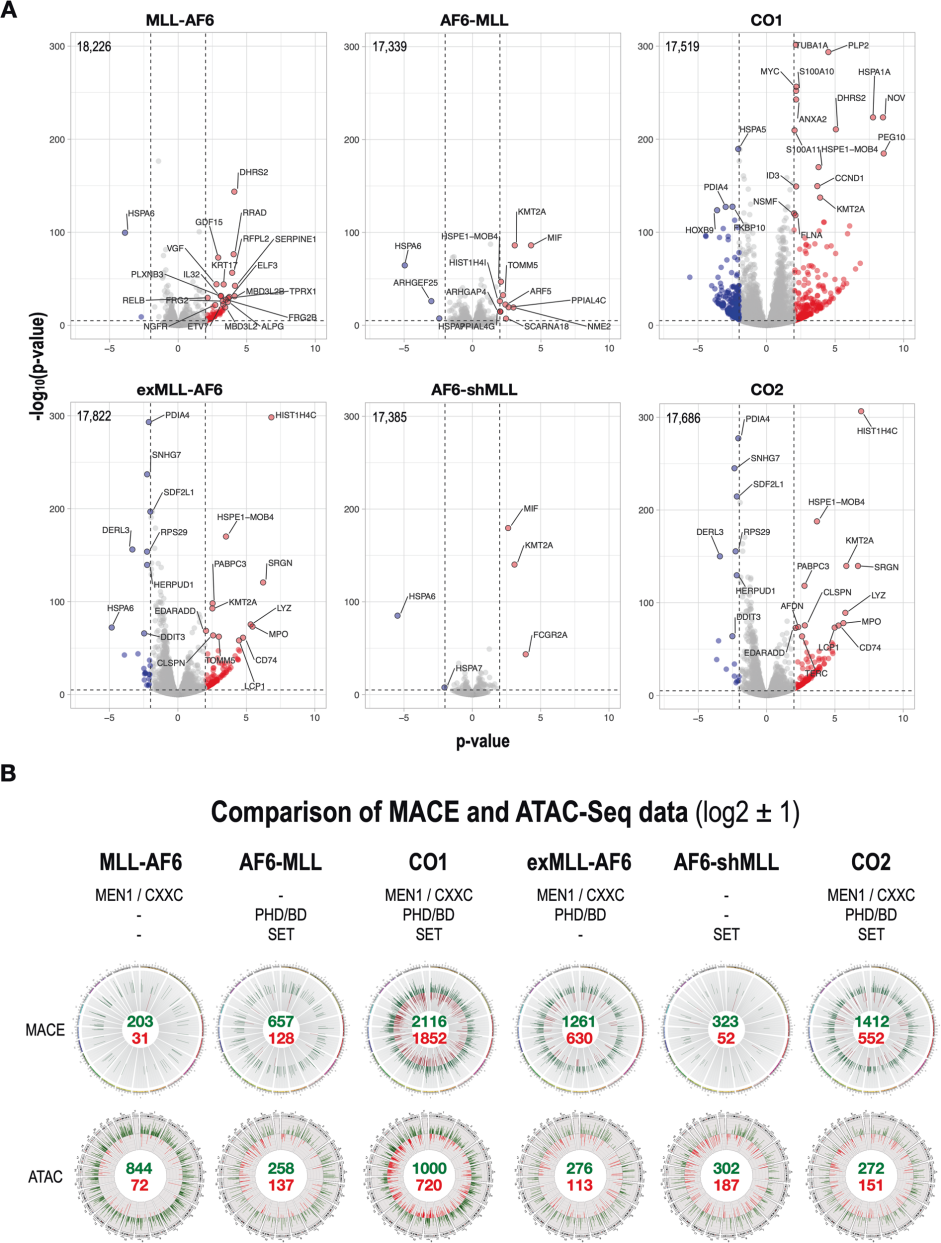
Volcano and Circos plot analysis of the two data sets of t(6;11) fusion genes. **A** Gene entries for protein-coding genes of all six cell lines were used to visualize the significant changes by volcano plots. Gene symbols together with *p* values, log2 changes and - log10 (*p* value) data were used of each cell line to perform the analyses (VolcaNoseR website, huygens.science.uva.nl). The number of gene entries used for the plots is displayed in the top left corner. We used very stringent parameters to visualize in red and in blue the most significant changes in gene expression (log2 = ±2, −log_10_(*p* value) > 5). Also, here a significant up- and downregulation are only seen on CO1 cells, while the patterns in exMLL-AF6 and CO2 cells are nearly identical and display mostly upregulated genes. **B** The identified signatures deriving from MACE- and ATAC-Seq experiments were used to create Circos plots in order to help to interpret and understand the findings for each of the six cell lines. Green and red numbers display the number of up-and down-regulated genes with a log2 value of ±1. Similarly, the numbers of increased and decreased chromatin accessibility of the ATAC-Seq experiments are shown for all chromatin fragments that displayed a *p* value of smaller than 0.05. The comparison between ATAC-Seq and MACE-data allows to intuitively understand the actions of single MLL fusion proteins, as well as of their co-expression. Again, CO1 and CO2 cells displayed the highest numbers of deregulated genes or differential chromatin accessibility. Above the Circos plots, important protein domains are displayed that are present by the fusion proteins in each of the six cell lines (MEN1, CXXC, PHD/BD, and SET).

Similarly, we performed Volcano plot analyses with the protein coding genes sets that are summarized in Fig. 3A. The total number of gene entries representing the protein coding genes is indicated for each plot. Of interest, *MLL/KMT2A* is one of the top-scoring genes that could be identified in all cell lines (MLL-AF6 FC = 1.5, AF6-MLL FC = 9.1, CO1 FC = 16.4, exMLL-AF6 FC = 6.1, AF6-shMLL FC = 9.1 and CO2 FC = 64.6). Since the MLL-C-terminus is part of our constructs in all reciprocal fusion constructs, this result may be explained as experimental artifact for the single transfected cell lines expressing AF6-MLL, AF6-shMLL or the co-expressing cell lines, CO1 and CO2, respectively. However, this explanation is not valid for exMLL-AF6 expressing cells, indicating that the endogenous *MLL* gene is a direct target of the exMLL-AF6 fusion protein. Another interesting finding is the *MIF* gene that can only be found in the cells expressing the PHD/BD domain. High MIF expression (Macrophage Inhibitory Factor) has been recently linked to worse outcome and high relapse in leukemia patients (see discussion). As a last example, the *MPO* gene—a classical myeloid-specific genes—was only seen in cells expressing the exMLL-AF6 fusion protein. Based on these analyses, we concluded that both sets of fusion protein exhibited very different molecular mechanisms in our model system.

### Analyzing the shared and idiosyncratic gene signatures of CO1 and CO2 cells

Next, we analyzed the obtained protein coding gene signatures of CO1 and CO2 cells for their common and idiosyncratic gene expression (up- and down-regulated genes). As summarized in Fig. S3, CO1 and CO2 cells display 266 up- and 10 commonly downregulated protein-coding genes. A subsequent pathway analyses revealed that the upregulated signature is attributed to “cellular developmental processes”, “immune system processes”, “cell activation” and “positive regulation of molecular functions”, while the downregulated signature has a link to the “relieved ER stress pathway”. More importantly, the idiosyncratic signature of CO1 cells display links to “cellular developmental processes”, “animal organ development”, “regulation of developmental processes” and “regulation of transcription by RNA polymerase II”, as well as the “regulation of cell differentiation”, while the downregulated signature of CO1 cells shows similar pathways including “nervous system development” and “embryo development”. The idiosyncratic signature of upregulated genes (*n* = 135) in CO2 cells revealed a large set of genes that can be attributed to “lymphoid cells” with several well-known T-cell markers, such as CD4, CD75, LAT2, IKZF1, LMO2. The identification of such a T-cell signature in HEK293 cells was unexpected. The downregulated idiosyncratic signature in CO2 revealed no pathway, most likely due to the small number of protein-coding genes in this signature.

### Chromosome usage analysis revealed patterns attributing the pathomolecular power of the different t(6;11) fusion genes

Next, we analyzed the datasets with the GUDC module, as depicted in Fig. S4. By simply examining these fingerprints, it became intuitively clear that MLL-AF6 and AF6-MLL together were changing the gene expression on all 22 chromosomes and the X chromosome. The strongest effects were observed when both fusion proteins were expressed (CO1) and resulted in strong deviations seen on the X chromosome, followed by chromosomes 21, 7, 19, 8, and 9. Vice versa, the most downregulated genes were found again on chromosome X, followed by chromosomes 17, 18, 16, and 10. Vice versa, the exMLL-AF6 fusion protein alone is mainly responsible for the changes seen in gene expression patterns, which was nearly identical in CO2 cells. Here, the chromosome pattern displays the strongest upregulation of genes that are localized on chromosomes 13, 12, 7, and X. A significant pattern for downregulation was hardly visible, and if any, these genes were localized on chromosomes 22, 4 and 12.

### Comparison of the MACE and ATAC-Seq data revealed different target genes affected by t(6;11) fusion proteins

The ATAC-Seq experiment revealed the accessible or non-accessible chromatin fractions in all 6 cell lines when compared to the equally treated mock-cell line. The resulting chromatin signatures had quite similar mean reads/gene (Fig. S5, upper panel). All these gene entries were first analyzed for accessible (log2 value > 0) and non-accessible fractions (log2 value < 0) genes. All data entries were then filtered to select target-gene signatures (>2 reads, *p* value < 0.05 and FC > ±2 or ±4). Also here the DAGT module allow to classify identified chromatin regions associated with pseudogenes, non-annotated genes, LincRNA genes, microRNA genes, SNO genes, mitochondrial genes and protein-coding genes. These data were then displayed by Circos plots which were then compared to the Circos plots deriving from the different MACE experiments. In Fig. 3B, MACE-Seq and ATAC-Seq are shown for the obtained signatures with both sets of t(6;11) fusion proteins. Form this comparison it became obvious that although MLL-AF6 appears to make the chromatin more accessible, only a few genes were up or downregulated. AF6-MLL seems to induce also some changes in the chromatin, but the resulting gene expression signature is more than double of that with MLL-AF6 alone. CO1 cells displayed the strongest changes in chromatin accessibility and gene transcription. By contrast, the two cell lines AF6-MLL and CO1 displayed a much higher number of deregulated genes as could be anticipated by the observed changes in the chromatin. This is an important observation, as it may suggest that the presence of the reciprocal fusion protein may allow deregulating genes—independent from the chromatin status. The second set of t(6;11) fusion proteins shows similar effects when the exMLL-AF6 protein is present (exMLL-AF6 or CO2 cells), while AF6-shMLL had only limited impact. Thus, we concluded that the presence of the PHD/BD domain has a quite important function, namely, to enable deregulated gene expression independent from the chromatin status.

In order to validate this assumption in more detail, we carefully analyzed the six ATAC data sets and compared them to the data sets obtained by the MACE experiments (Fig. S6). In this figure we dissected the obtained ATAC signatures according to the different gene types (pseudogenes/non-annotated genes (PG/NA) vs. protein coding genes (PCG)) and evaluated the comparability of the ATAC and MACE signatures. MLL-AF6 activated dominantly protein-coding genes (*n* = 146) from active chromatin fractions, while downregulated genes (*n* = 30) could be attributed to less active chromatin fractions. This situation changed in a dramatic fashion we analyzed AF6-MLL expressing cells. Here, most activated genes were classified as PG/NA genes (*n* = 565) which nearly equally derived from active and inactive chromatin fraction. In co-transfected cells most activated genes belonged to the PG/NA fraction (*N* = 1192) of which 2/3 derived from active chromatin while 1/3 from inactive chromatin fractions. Most downregulated genes were classified as PCG’s that could be associated to ~2/3 with inactive chromatin and ~1/3 with active chromatin.

When analyzing the second set of t(6;11) fusion proteins, the exMLL-AF6 fusion protein alone already activated many PG/NA genes. In particular, the amount of PCG’s was nearly triplicated (146->404), but the amount of PG/NA genes was about 20-fold higher (42->816). This was also true for the downregulated target genes (12->260 PC/NA genes; 18->354 PCGs). The target gene spectrum of the reciprocal fusion protein AF6-shMLL was reduced to roughly 50% and equally distributed to active and less active chromatin fractions. Finally, CO2 cells display more less the pattern from exMLL-AF6 cells, and also here, a clear link of active genes deriving from to active chromatin, or, inactive genes associated with inactive chromatin fractions was nearly lost. In conclusion, the fusion proteins expressed in cells with AF6-MLL, exMLL-AF6, AF6-shMLL, and CO2 appear to deregulate their target genes nearly equally from accessible and non-accessible chromatin regions.

This led us to the conclusion that both sets of fusion genes exert a different mode of action. In particular, the presence of the PHD/BD domain appears critical for the function of the fusion proteins, as it allows them to activate specifically the group of non-annotated genes and pseudogenes (see Fig. 3B, domains above the circos plots). The combination of physically separated MEN1-binding/CXXC domain and PHD/BD/SET domain appear to have the strongest impact on changes in chromatin and gene expression. Thus, our analyses were able to attribute distinct molecular consequences to certain protein domains.

### Analyzing the de novo genes and shut-down genes by the DAGE module revealed a highly important gene signature

**Fig. 4:**
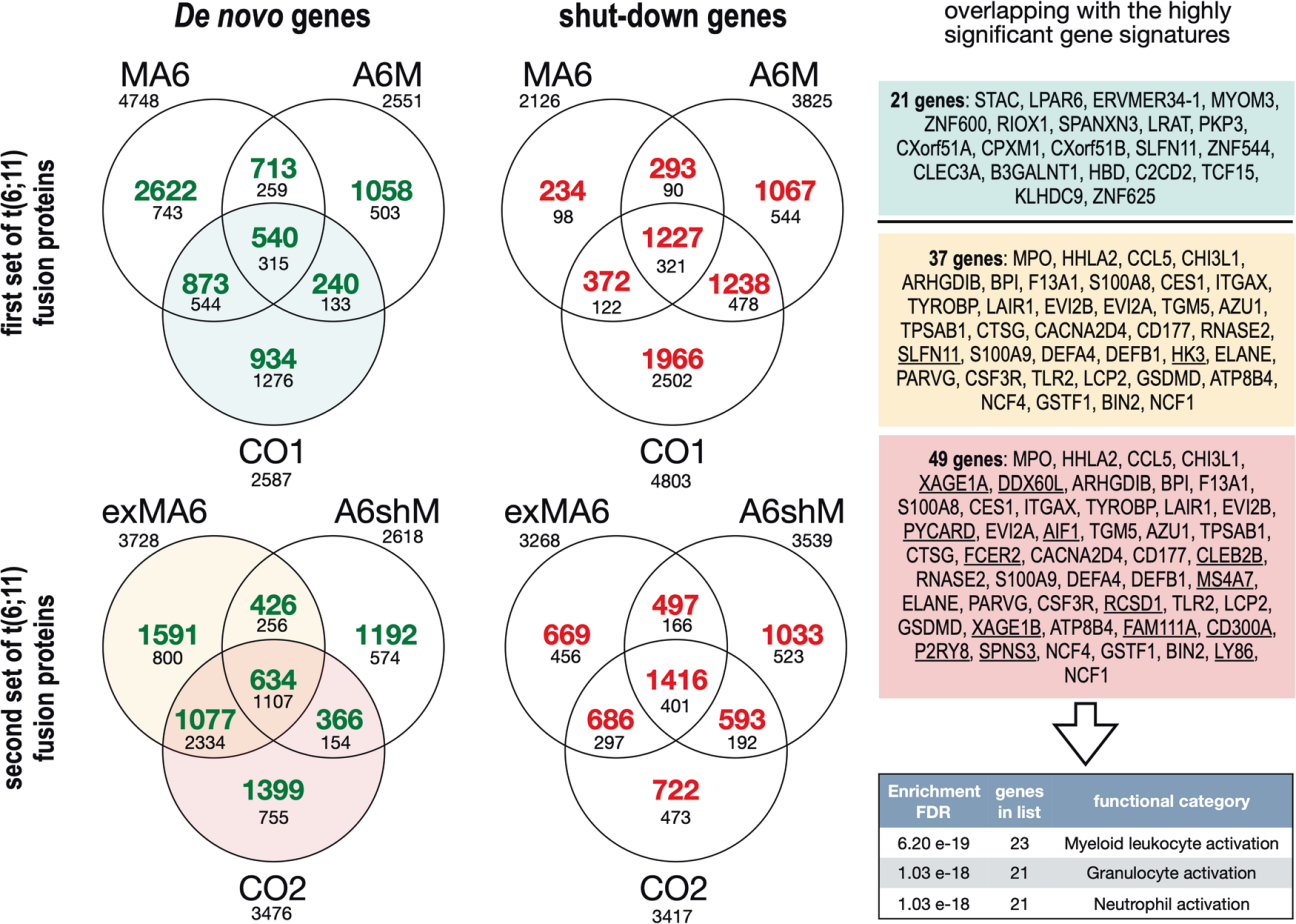
Analyses of de novo and gene shut-down by the DAGE module. The Filemaker Database program was used to identify reliably all genes that were either de novo induced or were completely shut-down due to the presence of fusion proteins. The resulting VENN diagrams show the distribution of the identified target genes by number, and which target gene is induced or repressed by which fusion protein. Along with the number of genes (colored in green or red), the “number of reads” for these gene sets is displayed; in case of upregulation, the reads of the de novo genes is displayed; in case of downregulation, the displayed number reads are deriving from the mock cell line. Certain areas in the VENN diagrams were colored in light blue, light orange and light red. These parts of the VENN diagrams correspond with the right colored areas, where we have displayed gene names. The genes which we displayed were overlapping between de novo upregulated genes and the highly significant gene sets identified to be at least 4-fold upregulated (980 genes for CO1 cells, 608 genes for exMLL-AF6, and 655 for CO2 cells). The 21 highly upregulated genes found in CO1 cells (light blue) did not reveal a convincing pathway, however, the genes displayed in the light orange (37 genes) and red boxes (49 genes) overlapped nearly completely. The two underlined gene names in the light orange box derived from exMAF6, all genes not underlined in the light orange and red box (*n* = 35) derived from the intersection, and the 14 underlined gene names in the light red box derived from CO2 cells. Of interest, these few genes revealed a pathway-specific for myeloid cells.

Finally, we investigated de novo gene expression, as well as the shut-down gene transcription in the six different signatures by the DAGE module. As shown in Fig. 4, several thousand genes became de novo activated or shut-down in the presence of single or both fusion protein pairs (upregulated genes: green, downregulated genes: red). The VENN diagrams also highlight the overlaps between the different settings. Most of these genes are barely expressed when the number of reads was analyzed (shown as black numbers).

The surprise came when we compared these signatures with our highly significant signatures shown in Fig. 2A, because CO1 overlapped with 21, exMLL-AF6 overlapped with 37, and CO2 with 49 protein-coding genes. The signature in CO1 cells points to the several pathways (vision: diseases of neuronal pathways and retinoid metabolism), while the signatures deriving from exMLL-AF6 and CO2 cells were overlapping with the 3 pathways “innate immune cells”, with a clear gene signature pointing to myeloid cells (granulocytes and neutrophils). This led us to the conclusion that exMLL-AF6, alone or in combination with AF6-shMLL, can able to turn on a myeloid-specific genetic program in these stably transfected cells.

Taken together with the identified T-cell specific gene signatures in CO2 cells (Fig. S3) it demonstrated the instructiveness of these t(6;11) fusion proteins, even when expressed in a test model system that is far away from the hematopoietic cells.

## Discussion

Here, we present the pathomolecular relevance of direct and reciprocal fusion proteins deriving from the major (introns 9–11) or minor breakpoint cluster region (introns 21–23) of the *MLL* gene. The particular interest to investigate these t (6;11) fusion proteins came from the clinical observation that leukemia patients with breakpoints in the major BCR are mostly diagnosed with AML, while patients with breakpoints in the minor BCR are exclusively diagnosed with T-ALL.

The data obtained by the MACE-Seq experiments revealed the potential of each fusion protein to deregulate gene transcription (Fig. 2A). When comparing the gene signatures of MLL-AF6 and CO1 cells in more detail, we observed roughly 12-times more upregulated genes when both fusion genes, MLL-AF6 and AF6-MLL, are expressed together (88 vs. 980). In addition, downregulation of genes became enabled (2 vs 480). This clearly argued for a strong cooperativity of both fusion proteins, resulting in a massive amplification of deregulated target genes. This picture changed when we analyzed the second set of fusion proteins. Expression of the direct exMLL-AF6 fusion protein alone resulted already in a large signature of 691 highly deregulated genes (608 up- and 83 down-regulated genes), while the reciprocal fusion AF6-shMLL did not play a major role. However, the CO2 cells had slightly more deregulated genes, indicating that the reciprocal construct contributed in an additive fashion. Noteworthy, the observed differences for the 2 sets of t(6;11) fusion proteins seem to depend only on the swapped PHD/BD domain (from AF6-MLL to exMLL-AF6).

All these findings were further analyzed by heatmap analyses (Fig. 2B), volcano plot analyses (Fig. 3A), pathway analyses (Fig. S3), or the results when using the the three analytical modules. Co-expression of MLL-AF6 and AF6-MLL caused also a large set of significantly down-regulated genes, a phenomenon which was nearly absent in the second set of t(6;11) fusion proteins (Fig. S4). A putative explanation could be the very strong activation of the endogenous *MLL* gene, because we did see very strong signals also for the *MLL* gene in the ATAC-Seq data (mock: 32 reads; MLL-AF6: 193 reads; AF6-MLL: 1,171 reads; CO1: 441 reads; exMLL-AF6: 143 reads; AF6-shMLL: 1444 reads; CO2: 2099 reads). These data clearly show that *MLL* gene loci were much more associated with activated chromatin and more strongly expressed in cells expressing the second set of t(6;11) fusion proteins.

Since MLL protein is highly expressed in developing tissues, we were not so much surprised to find a T-cell-specific signature in CO2 cells (Fig. S3). Together with the myeloid gene signature that we could identify in the most prominent transcribed de novo genes (Fig. 4), we have to conclude that the second set of t(6;11) fusion proteins was indeed able to mimic somehow the myeloid and T-cell specific phenotype that is already known from human t(6;11) leukemic cells.

The volcano plot analyses revealed the *MLL* and *MPO* as potential target genes of the exMLL-AF6 protein which was highest activated in CO2 cells. The identified *MIF* gene was only highly activated in cells that express fusion proteins that exhibit the PHD/BD domain. MIF has been recently identified as a critical target correlated with a worse outcome in leukemia patients, as it was defined as an independent prognostic factor important for OS and DSF [39].

The Circos plots (Fig. 3B) revealed another important finding when comparing MACE- with ATAC-Seq data: the importance of the PHD/BD domain. The presence of the PHD/BD domain in a given fusion protein (AF6-MLL or exMLL-AF6) allows to significantly deregulate more genes than anticipated from the investigated chromatin status. This unusual phenomenon is also visible in CO1 and CO2 cells which express also the above-mentioned PHD/BD-exhibiting fusion proteins. In both cases, up and down-regulated genes were deriving equally from active and less active chromatin fractions, indicating for an important feature of this domain to recruit target genes by a yet unknown mechanism. It has been shown in the past, that wildtype MLL is recruited to target genes via the CXXC and PHD/BD domain [21]. The CXXC domain is important because of PAF1 interactions, while the third PHD finger of the PHD/BD domain was required to read the H3K4_me2/3_ chromatin signatures at target gene loci. Whether wildtype MLL can be recruited to target genes by only the PHD/BD domain is yet unclear, but the fact the exMLL-AF6 exhibits both domains (CXXC and PHD/BD) may provide an explanation for the differences in up-and down-regulated genes observed in CO1 and CO2 cells. Another explanation could derive from the possibility that fusion protein cooperates with endogenous MLL protein, and this could be again due to the presence of the PHD/BD domain, which has been described in the past as protein-protein interaction domain for MLL itself [40].

One of the phenomenon’s associated with the expression of AF6-MLL or exMLL-AF6 (also in CO1 and CO2 cells) was the strong activation of pseudo- and non-annotated genes (Fig. 2A). This strong increase in pseudo- and non-annotated genes is a mechanistic hint for an increased oncogenic potential, because these genes were already shown to provide benefits for malignant cell growth [41]. This was nicely visible in the comparison analysis of MACE- and ATAC-Seq data (Fig. S6, left table). When looking to the upregulated genes (marked in green) the highest number of pseudogenes/non-annotated genes was found in CO1 cells (*n* = 1192), followed by CO2 (*n* = 841), exMLL-AF6 (*n* = 816) and AF6-MLL cells (*n* = 595). When looking to the red-marked downregulated gene section, then all cell lines displayed a much lower rate of pseudogenes/non-annotated genes. The highest amount of pseudogene/non-annotated genes was found in exMLL-AF6 cells (*n* = 260), followed by CO1 (*n* = 253) and CO2 cells (*n* = 248), while the ratio between PG/NA vs. PCG was lowest in CO1 cells (16%) due to the dramatic amount of down-regulated protein coding genes (*n* = 1558). Again, a strong upregulation of PG/NA genes was always visible in all cell lines that expressed a fusion protein exhibiting the PHD/BD domain. In the downregulated signatures, the PCG’s always outnumbered the amount of downregulated PG/NA genes.

The comparative analyses of MACE- and ATAC-Seq data allowed to draw a second conclusion: while MLL-AF6 alone generated its gene signature mainly from already existing active chromatin, the presence of the reciprocal fusion protein allowed the deregulation of target genes regardless of whether they were present in more accessible or less accessible chromatin (Fig. 3B, AF6-MLL or CO1). This result gives a first glimpse on an important potential function of reciprocal MLL fusions (containing a PHD/BD domain), namely, to allow the activation or repression of genes without changing the general chromatin condition in their vicinity. A similar observation has been made in the past also for the reciprocal AF4-MLL fusion protein that was designated as a “chromatin opener” in a similar context [42–44]. If so, the presence of reciprocal MLL fusion proteins would allow a given direct MLL fusion protein to use the genome in an adaptive way to cope with different situations.

Since both sets of fusion proteins differ only in the presence or absence of the PHD/BD domain, this raises new questions about the functions deriving from this particular domain of the MLL protein (including other MLL family members or proteins that harbor such PHD domains). So far, the PHD/BD domain is known as a molecular trigger when binding to the CYP33 Isoprolylisomerase [40]. This trigger toggles between being a chromatin reader domain or to allow recruitment of a BMI1 repressor complex to the CXXC domain [24, 44–46]. This is of course only possible in the wild-type MLL protein, but also in the exMLL-AF6 fusion protein, but not in the MLL-AF6 fusion. The BD domain itself is not a functional bromodomain, rather it helps to stabilize the PHD3 reader domain. In addition, other groups have already shown that the three different PHD domains also control protein maintenance because it binds to two different E3-ligases that control proteasomal degradation [47, 48].

Taken together all these data, we do believe that we have identified a key mechanism that can be attributed to the initial pathway that finally leads to *MLL-r* leukemia. We pose the hypothesis that the disruption of the MLL protein between the CXXC domain and the PHD/BD domain causes a dramatic effect: it results in a direct fusion protein that is able to strongly enhance target gene transcription, but the additional presence of a complementary, reciprocal fusion protein enables the use many other genes encoded by the genome that are usually not available for gene transcription. Such an “adaptive genome usage” could be important, as it allows a given cell to change rapidly its cell fate in a Lamarckist process of adaptation. These novel features make a pre-tumor cell nearly omnipotent with regard to the “use of genes”. Over time and depending on external signals, it will convert a normal cell into an aberrant cell, and most likely causes the onset of cancer, combined with strong features of pluripotency. This is presumably one of the best definitions we can make for the most commonly occurring *MLL-r* leukemias known today.

A T-cell specific gene signature (Fig. S3), was only seen with fusion proteins deriving from the minor BCR of MLL. Together with the myeloid gene signature that we could identify in the most prominent transcribing de novo genes (Fig. 4), we have to conclude that the second set of t(6;11) fusion proteins was indeed able to mimic the myeloid/T-cell-specific phenotype that is already known from human t(6;11) leukemic cells.

Thus, we believe that we have shed light on the molecular mechanism that defines preleukemic cells, as such MLL fusion proteins require only 48 h to make a dramatic change in the genome-wide landscape of a given cell.

## Methods

### Cell culture and transfections

HEK293T cells were grown in DMEM with 10% (v/v) FCS (Capricon Scientific), 2 mM L-Glutamine (Capricon Scientific), and 1% (v/v) Pen Strep (GE Healthcare) at 37 °C and 5% CO_2._ The single (*n* = 4) and co-transfected (*n* = 2) stable cell lines from all the above-mentioned constructs were established by using low amount (50 ng) of SB transposase vector SB100X. Metafectamine mediated transfection into HEK293T cells were carried out as recommended by the manufacturer (Biontex). After 24 hours, cells were subjected to Puromycin (1 µg/ml) or Blasticidin (15 µg/ml) or both for selection. The cells were incubated with selection markers for 3–10 days and terminated when virtually all cells were emitting the expected green or red color derived from their corresponding reporter genes (eGFP or dTom respectively). The cells were further cultivated for 4 weeks without selection markers and the stability of transfected vector constructs was monitored. In all cases, the transfected cells remained stable, expressing their respective reporter and selection marker.

### RNA extraction, cDNA synthesis, and RT-PCR experiments

The transgenes were induced by using 1 µg/ml Doxycyclin to the cell culture for 48 h. Afterward, total RNA was isolated using RNeasy^®^ Mini Kit (Qiagen) and cDNA synthesis was performed using SuperScript^®^ II (Invitrogen). All isolated RNAs were quality checked (Agilent Bioanalyzer) and final concentrations were determined. Equal amounts of total RNA were used throughout all experiments. All primers used for RT-PCR analyses are listed as follows: MLLe8F (5′-ACCTACTACAGGACCGCCAA-3′), MLLe10R (5′-TCTGATCCTGTGGACTCCAT-3′), MLLe12F (5′-GCAAATTCTGTCACGTTTGT-3′, MLLe15R (5′-TTGTCACAGAGAGGGCAGAAGTT-3′), MLLe23R (5′-GGTGCAGGATGTGAGACAGCA-3′), AF6e1F (5′-GGCCGACATCATCCACCACT-3′), AF6e2R (5′-GAAATTTCTCCGCGAGCGTTT-3′), MLLe20F (5′-AGACTCACCAACTCCTCTGC-3′). With these oligonucleotides, all splice events within the 4 vector constructs were tested. The resulting PCR fragments were run on 1% agarose gels and subsequently subjected to DNA-sequencing analysis to validate all splicing events were correctly executed.

### Differential gene expression profiling by MACE-Seq

The chimeric genes were induced for 48 h with 1 µg/ml Doxycyclin and total RNA was isolated from transfected cell lines. In order to validate correct transgene expression, the following primers were used for RT-PCR analyses: MLL8.3 (5′-CCCAAAACCACTCCTAGTGAG-3′), MLL13.5 (5′-CAGGGTGATAGCTGTTTCGG-3′), MLL21.3 (5′-GTCGACAAGACAGTCCAGAGC-3′), MLL26.5 (5′-TGGTGCTCCAGTATACCCTGG-3′), AF61.3 (5′-TCGAGATCAGCCAGCCGACC-3′) and AF65.5 (5′-GTAAACCTCAGCAGCCAGTCG-3′). After testing the correct induced expression of all transgenes, differential gene expression (DGE) profiles were obtained by MACE (Massive Analysis of cDNA Ends)—Seq (Sequencing) following the manufacturer protocol (The MACE-Seq Kit, GenXPro, Frankfurt, Germany). Resulting data from three biological replicates of all six cell lines were compared with three biological replicates of mock-transfected cells. All data were analyzed by DESeq2 and resulting output data were implemented in the database program FileMaker for further analysis. All the raw data have been submitted to the NCBI GEO server where these data can be retrieved by the following accession codes: GSE17558 (ATAQ-Seq data) and GSE175573 (MACE-Seq data).

### ATAC sequencing experiments

Preparation of ATAC samples was performed according to a published protocol [49]. Further details are described in the Supplementary data file.

## Supplementary information

Supplemental Data file

MACE-Seq MLL-AF6

MACE-Seq AF6-MLL

MACE-Seq MLL-AF6/AF6-MLL

MACE-Seq exMLL-AF6

MACE-Seq AF6-shMLL

MACE-Seq exMLL-AF6/AF6-shMLL

ATAC-Seq MLL-AF6

ATAC-Seq AF6-MLL

ATAC-Seq MLL-AF6/AF6-MLL

ATAC-Seq exMLL-AF6

ATAC-Seq AF6-shMLL

ATAC-Seq exMLL-AF6/AF6-shMLL

MACE Heatmap dataset 1

MACE Heatmap dataset 2
