# Supplementary material for: The role of reciprocal fusions in MLL-r acute leukemia: studying the chromosomal translocation t(6;11)
Source: Oncogene. 2021 Aug 5;40(40):5902–12. doi: 10.1038/s41388-021-01983-3 (PMC8497272; doi:10.1038/s41388-021-01983-3)

Supplemental Data file

Supplemental Excel files

The first set of 6 files (MACE-data) contain the gene entries with gene identifier, gene symbol, gene name, mean exp, mean mock, fold change log2, pvalue, -log10(pvalue), var-log2, chromosome number (with beginning and end), classification (into pseudogene (PG), non-annotated gene (NA), LINC RNA gene (LINC), MIR gene (MIR), SNO gene (SNO), MT gene (MT) and protein coding gene (PCG)). All data are given as in the Bioconductor output file with the exception of var-log2 which was simply calculated by ln(mean exp/mean mock)/ln(2). The following files are provided:

1. MLL-AF6 gene signature.xlsx
2. AF6-MLL gene signature.xlsx
3. CO1 gene signature.xlsx
4. exMLL-AF6 gene signature.xlsx
5. AF6-shMLL gene signature.xlsx
6. CO2 gene signature.xlsx

The second set of 6 files (ATAQ-data) contain the gene entries with gene identifier, gene symbol, gene name, mean exp, mean mock, fold change log2, pvalue, var-log2, chromosome number (with beginning and end), classification (into pseudogene (PG), non-annotated gene (NA), LINC RNA gene (LINC), MIR gene (MIR), SNO gene (SNO), MT gene (MT) and protein coding gene (PCG)). All data are given as in the Bioconductor output file with the exception of var-log2 which was simply calculated by ln(mean exp/mean mock)/ln(2). The following files are provided:

7. ATAC MLL-AF6.xlsx
8. ATAC AF6-MLL.xlsx
9. ATAC CO1.xlsx
10. ATAC exMLL-AF6.xlsx
11. ATAC AF6-shMLL.xlsx
12. ATAC CO2.xlsx

Additional Excel files provided are the gene sets used for the heatmaps:

13. Heatmap Set 1
14. Heatmap Set 2

Methods

QPCR experiments

Total RNA was extracted using RNeasy® Mini Kit (Qiagen). Complementary DNA was synthesized using SuperScript II reverse transcriptase (Invitrogen). An aliquot of the produced cDNA was used for quantitative PCR. Quantitative PCR were performed using ORA™ qPCR Probe ROX H Mix (highQu). Fluorescence signals above the threshold (CT) were determined with the aid of the StepOnePlus™ Real-Time PCR System (Life Technologies, Darmstadt, Germany). The fluorescence intensity of the samples was normalized to the amplification value of the reference gene glyceraldehyde-3-phosphate dehydrogenase (GAPDH). $2^{-\Delta\Delta CT}$ method was used to calculate the expression of the target mRNAs. For qPCR, the following primers were used: CCND1_For (5'-GCAAGGCCTGAACCTGAGG-3') in combination with CCND1_Rev (5'-CAGGCTTGACTCCAGCAGG-3'), KLF8_For (5'-CTGGGATGGCTGCTCC-TGG-3') with KLF8_Rev (5'-AGGGACAGGTGGTCAGAACG-3'), ZNF544_ For (5'-GGAAGC-ACGTTCTATGCTGG-3') with ZNF544_Rev (5'-CACCTCTCGGTACAGTGTCC-3'), NOV_For

(5’-ACCGTCAATGTGAGATGCTG-3’) with NOV_Rev (5’-CAGTTCTTGAACTGCAGGTGG-3’), PEG10_For (5’-CCCCATCCTTCCTGTCTTCG-3’) with PEG10_Rev (5’-GAGACCTCCC-AGCTGTAGC-3’), CD44_F (5’-ATCTTGGCATCCCTCTTGG-3’) with CD44_R (5’-GTCCAC-TTGGCTTTCTGTCC-3’), and GAPDH.3 (5’-CTTCACCACCATGGAGGAAGG-3’) with GAPDH.5 (5’-CCTGCTTCACCACCTTCTTG-3’)

Bioinformatic analyses and MACE-Seq experiments

All data received from the Bioconductor software from our RNA-Seq or ATAC-Seq experiments were incorporated into a FILEMAKER database program which allowed filtering the data, and cross-comparison analyses between different data sets. Moreover, gene ontology and other databases were implemented as well to retrieve the necessary information that has been used throughout this manuscript. Resulting data from 3 biological replicates of all cell lines were compared with 3 biological replicates of mock-transfected cells. The MACE-libraries were prepared at GenXPro GmbH using the Massive Analysis of cDNA Ends (MACE-Seq) Library Preparation Kit (v2.0) from GenXPro GmbH. Briefly, RNA was fragmented and and cDNA was generated using Oligo(dT) primers with distinct Oligo IDs per sample for subsequent pooling of up to 24 samples. After pooling, cDNA was fragmentated to an average size of 200 bp using the sonicator Biorupter Plus (Diagenode, Belgium). The distribution of cDNA fragment sizes was monitored using the automated microfluidic electrophoresis station LabChip GXII Touch HT platform (PerkinElmer, USA). The poly(A) containing cDNA fragments were purified using solid phase reversible immobilization (SPRI) beads (Agencourt AMPure XP, USA), end repaired and ligated to distinct 8-base pair UMI Adapters (also called TrueQuant adapters). Then, the library containing labelled and fragmentated cDNA was amplified by PCR, purified by SPRI beads (Agencourt AMPure XP, USA) and strand-specific sequenced using the HiSeq2500 (Illumina, USA). Bioinformatic analysis was performed according to the analysis pipeline for MACE libraries by GenXPro GmbH. Unique Oligo IDs and UMIs on each transcript allowed initial demultiplexing and subsequent removal of PCR-duplicates. The remaining reads were trimmed for high-quality as well as adapter-free sequences and aligned to the human reference genome (Genome Reference Consortium Human Build 38 patch release 13 (GRCh38.p13) using Bowtie 2. Resulting output data were implemented in the database program FileMaker for further analysis. All final data sets were exported from the FILEMAKER Database program as individual Excel documents for publication.

ATAC sequencing experiments

Cells were grown in 6-well plate and treated with 1 µg/ml Doxycyclin for 48 hours. After that cells were harvested, and viability was checked in every sample. In all samples viability was more than 90%. Cells were then resuspended in cold PBS and cell numbers in each sample were counted. 50,000 cells were centrifuged at 500 RCF for 5 minutes at 4°C in a fixed angle centrifuge. After centrifugation, 900 µl of supernatant was aspirated with P1000 pipette and the remaining 100 µl of supernatant was carefully aspirated by pipetting with a P200 pipette tip to carefully avoid the cell pellet. Cells were resuspended in 50 µl cold ATAC-Seq Resuspension Buffer (RSB; 10 mM Tris-HCl pH 7.4, 10 mM NaCl, and 3 mM $MgCl_2$ in water) containing 0.1% NP40, 0.1% Tween-20, and 0.01% Digitonin, pipetting up and down 3 times. Cells were incubated on ice for 3 minutes and then 1 ml of cold ATAC-Seq Resuspension Buffer (RSB) containing 0.1% Tween-20 but no NP-40 and Digitonin was added. Tubes were inverted 3 times to mix and then centrifuged at 500 RCF for 10 minutes at 4°C in a fixed angle centrifuge to pellet down the nuclei. Supernatant was removed with two

pipetting steps, as described before, and nuclei were resuspended in 50 µl of transposition mix (25 µl 2× TD buffer (20 mM Tris-HCl pH 7.4, 10 mM $MgCl_2$, 20 % Dimethyl Formamide in water), 2.5 µl transposase 26 (100 nM final), 16.5 µl PBS, 0.5 µl 1% digitonin, 0.5 µl 10% Tween-20, and 5 µl water) by pipetting up and down six times. Transposition reactions were incubated at 37°C for 30 min in a thermomixer with shaking at 1000 rpm. The final ATAC-Seq experiment was performed at GenXPro (https://genxpro.net/) that used an Illumina HiSeq for the analysis, and the resulting data were analysed by bioinformatics tools.

Figure legends

Figure S1: Workflow for the bioinformatic analyses

Upper panel: construction of the 6 stable cell lines together with the mock cell line. Transfection with Sleeping Beauty vectors usually took 7-12 days in order to obtain the selected and stable cell lines. Nucleic acids (RNA or DNA) were harvested 48h after the transgene expression was induced by 1 µg/ml Doxycyclin.Middle panel: MACE or ATAC-Seq experiments were performed and resulting data were analyzed by Bioconductor software to create output Excel files. Various other bioinformatic tools were used to analyze these data (volcano plots with huygens.science.uva.nl; heatmaps with biit.cs.ut.ee/clustvis; pathway analysis with bioinformatics.sdstate.edu). Circos plots have been used to display the genome-wide changes identified by RNA-Seq or ATAC Seq experiments. Finally, all Bioconductor output files have been imported to the Filemaker database program, which allowed to create novel modules for refined analyses not provide by the Bioconductor package (GUDC, DAGT, DAGE & ST). These modules allow to analyze the data beyond conventional data handling.

Figure S2: QPCR experiments with identified target genes

Target genes identified in CO1 cells by MACE were validated by QPCR experiments. Here, an example of 6 different analyses is shown. The QPCR data is shown for mock, MLL-AF6, AF6-MLL and CO1 cells, while the MACE reads are listed below. These analyses were made only for selected genes in order to demonstrate the high concordance between MACE-data and Q-RT-PCR data.

Figure S3: Detailed analyses of protein coding genes in the gene signatures of CO1 and CO2 cells

The identified gene signature in CO1 and CO2 cells were investigated for commonly and idiosyncratic protein coding genes. All these signatures (green colors: upregulated genes; red colors: downregulated genes). These subsets were then investigated by pathway analyses. CO1 (980 up- and 480 downregulated genes) and CO2 cells (655 up- and 74 downregulkated genes) share 256 protein coding genes, while displaying 243 and 135 idiosyncratic protein coding genes. The same data sets display 10 protein coding genes that were significantly downregulated, while having 341 and 8 protein coding genes downregulated as idiosyncratic signatures. Subsequently performed pathway analyses revealed interesting pathways which are shown by the different colors and identical numbers. Of interest, the up-regulated idiosyncratic signature of CO2 cells revealed a lymphoid-specific, and more specifically, a T-cell specific gene signature (see e.g. CD4, CD74, LAT2, IKZF1 and LMO2).

Figure S4: Signature analysis using to the GUDC module

Investigation of the distribution of activated and repressed genes on different chromosomes to generate patterns that demonstrate the genome-wide activity of the individual fusion proteins. The diagrams show two things: (1) mean transcription in percentage of genes located on all chromosomes (e.g. MLL-AF6 with 0,26% of all genes, while CO1 cells expressed 3,18% of all encoded genes); (2) the deviation from the mean transcription in the graph on the right. These

deviations display whether more or fewer genes became activated or repressed when compared to the mean of genes activated or downregulated on whole chromosomes. Thus, this module displays a fingerprint pattern of gene usage per chromosome by the individual fusion proteins. The analysis demonstrates again that effective up- and downregulation that occur only when MLL-AF6 and AF6-MLL are co-expressed in cells (CO1: synergistic effect), while exMLL-AF6 alone was capable to perform the upregulation of genes, while a down-regulation is nearly missing. The pattern only slightly changed in CO2 cells, arguing for an additive effect of both fusion proteins exMLL-AF6 and AF6-shMLL, respectively.

Figure S5: Dissection of the ATAC-Seq data

The obtained ATAC data from our experiments were summarized. Upper panel: left column displays names of all fusion genes or their combination (CO1 or CO2). The following columns contain information about number of gene entries and the number of totals up- and down-regulated genes. The last 4 columns represent the total reads of the up- and down-regulated gene signature. These were calculated for up- and downregulated chromatin regions by a minimum of 2 reads, a p-value < 0,05 combined with log2 changes of > 1 or 2 in case of up-regulated genes, while downregulated genes were identified by a minimum of 2 reads in the mock sample, a p-value < 0,05 combined with a log2 value of < -1 or -2. Lower panel: all data were dissected for the number of pseudogenes, non-annotated genes, LincRNA genes, microRNA genes, SNO genes, mitochondrial genes and protein coding genes, respectively.

Figure S6: Signature analysis using to the DAGT module

Cross-comparison between MACE and ATAC-data. Left table summarizes the gene signatures of all 6 cell lines with precise numbers for pseudogene/non-annotated genes (PG/NA) and protein coding genes (PCG) in the up- (green) or down-regulated gene signatures (red). Right graphic: the signatures of highly deregulated genes (log2 = ±1) found in the MACE experiments was compared to more or less accessible chromatin found in the ATAC-Seq experiment. Numbers in the green and red rectangles show e.g. that 198 out of 203 identified up-regulated genes in MLL-AF6 cells could be attributed to 151 accessible and 47 less accessible chromatin fragments. *Vice versa*, 31 out of 31 MACE downregulated genes could be associated with 9 accessible and 22 less accessible chromatin fragments. In addition, we analyzed the gene type distribution for all 4 possible scenarios. Below the Table, the circle plot explained. All signatures were subdivided into protein coding genes (PCG), SNO RNA genes (SNO), MIR genes (MIR), LINC genes (LINC), non-annotated genes (NA) and pseudogenes (PG). Since the most abundant gene types are protein coding genes, as well as the class of pseudogene/non-annotated genes, we summed up the latter two (pink numbers) and compared it to PCG´s (blue numbers) by indicating their percentages in the center of each circle plot. As an example, the 151 genes identified for the upregulated gene signature in MLL-AF6 cells derived and deriving from the accessible chromatin fraction (green rectangle) contained 72.2% PCG's but only 19,8% pseudogenes/non-annotated genes. This type of analysis was performed for all 24 subsections.

Figure S1

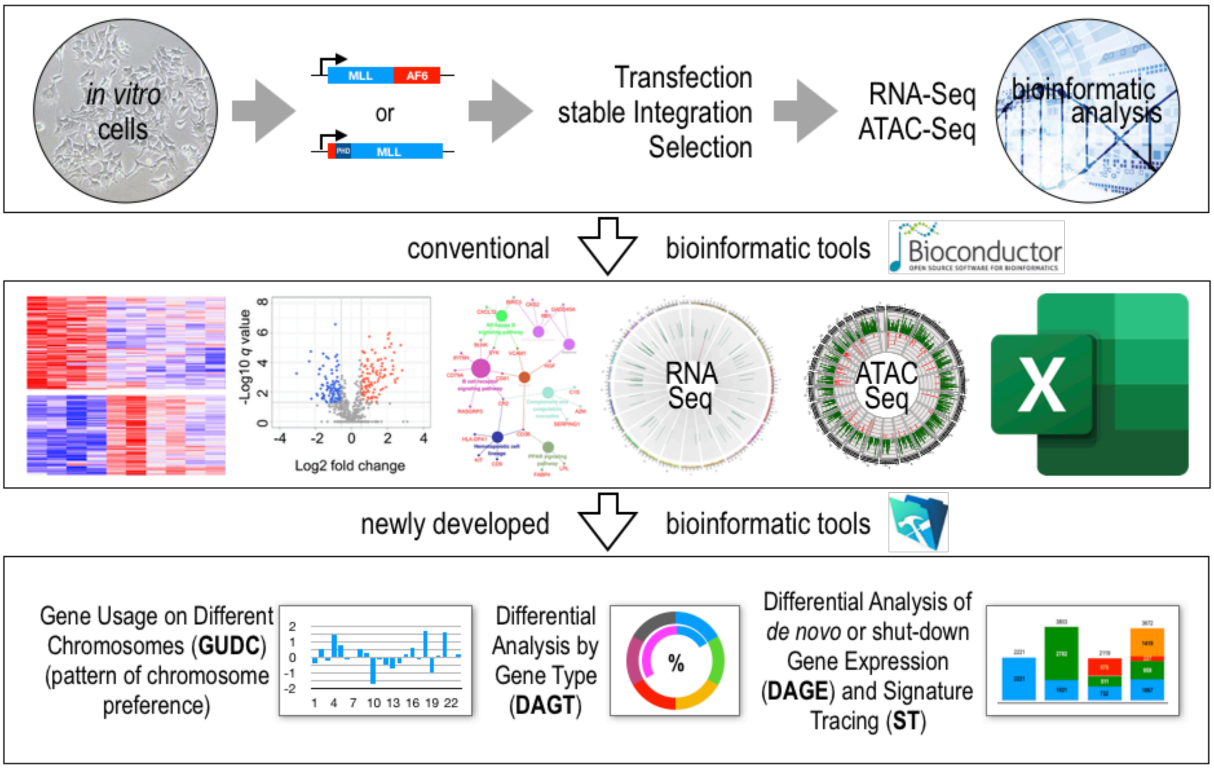

Figure S2

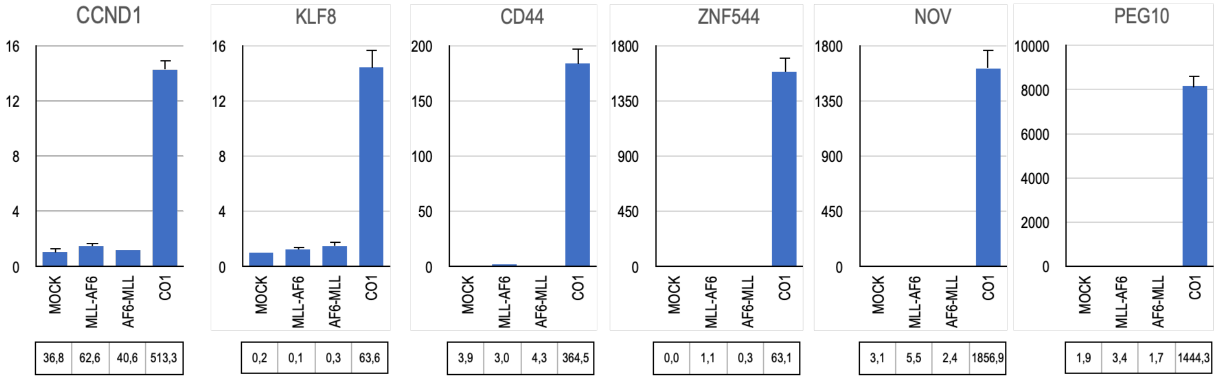

Figure S3

signature protein coding gene analysis

CO1
980 up
480 down

CO2
655 up
74 down

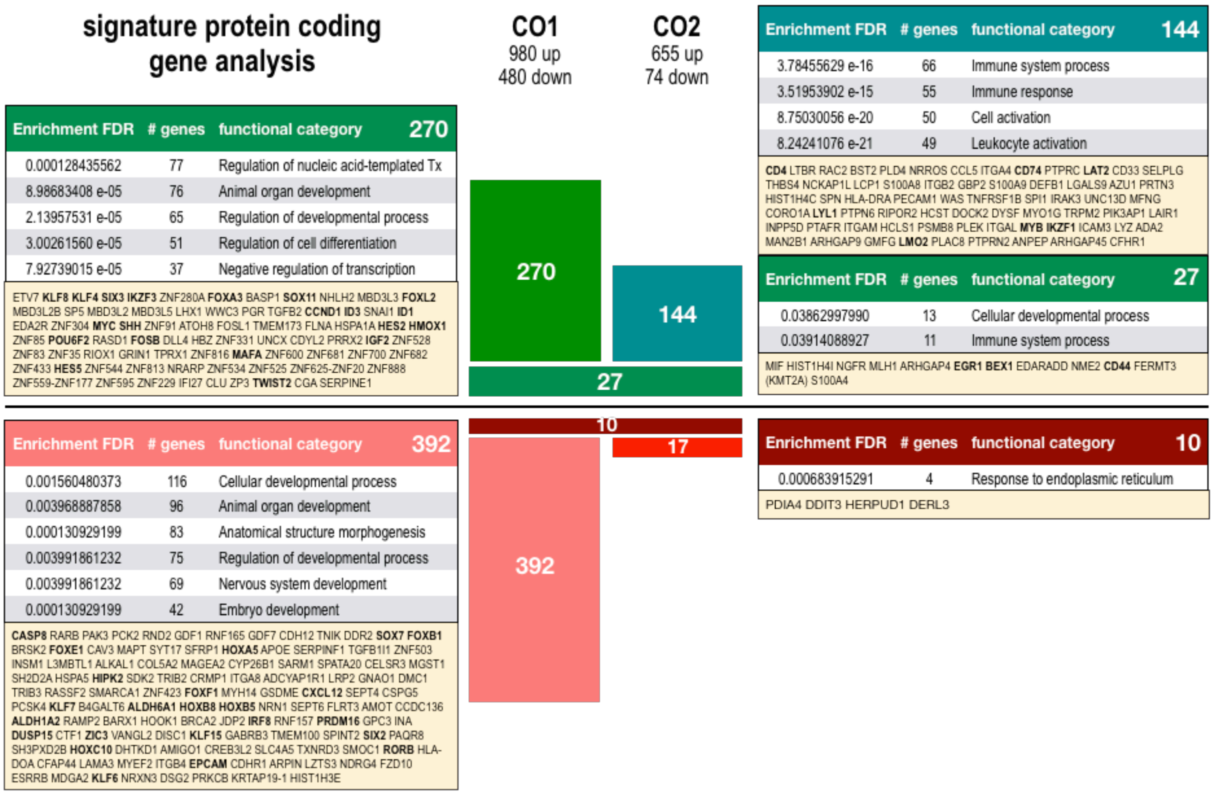

Enrichment FDR	# genes	functional category	270
0.000128435562	77	Regulation of nucleic acid-templated Tx	
8.98683408 e-05	76	Animal organ development	
2.13957531 e-05	65	Regulation of developmental process	
3.00261560 e-05	51	Regulation of cell differentiation	
7.92739015 e-05	37	Negative regulation of transcription	

ETV7 **KLF8 KLF4 SIX3 IKZF3** ZNF280A **FOXA3** BASP1 **SOX11** NHLH2 MBD3L3 **FOXL2** MBD3L2B SP5 MBD3L2 MBD3L5 LHX1 WWC3 PGR TGFB2 **CCND1 ID3** SNAI1 **ID1** EDA2R ZNF304 **MYC SHH** ZNF91 ATOH8 FOSL1 TMEM173 FLNA HSPA1A **HES2 HMOX1** ZNF85 **POU6F2** RASD1 **FOSB** DLL4 HBZ ZNF331 UNCX CDYL2 PRRX2 **IGF2** ZNF528 ZNF83 ZNF35 RIOX1 GRIN1 TPRX1 ZNF816 **MAFA** ZNF600 ZNF681 ZNF700 ZNF682 ZNF433 **HES5** ZNF544 ZNF813 NRARP ZNF534 ZNF525 ZNF625-ZNF20 ZNF888 ZNF559-ZNF177 ZNF595 ZNF229 IFI27 CLU ZP3 **TWIST2** CGA SERPINE1

Enrichment FDR	# genes	functional category	144
3.78455629 e-16	66	Immune system process	
3.51953902 e-15	55	Immune response	
8.75030056 e-20	50	Cell activation	
8.24241076 e-21	49	Leukocyte activation	

CD4 LTBR RAC2 BST2 PLD4 NRROS CCL5 ITGA4 **CD74** PTPRC **LAT2** CD33 SELPLG THBS4 NCKAP1L LCP1 S100A8 ITGB2 GBP2 S100A9 DEFB1 LGALS9 AZU1 PRTN3 HIST1H4C SPN HLA-DRA PECAM1 WAS TNFRSF1B SPI1 IRAK3 UNC13D MFNG CORO1A **LYL1** PTPN6 RIPOR2 HCST DOCK2 DYSF MYO1G TRPM2 PIK3AP1 LAIR1 INPP5D PTAFR ITGAM HCLS1 PSMB8 PLEK ITGAL **MYB IKZF1** ICAM3 LYZ ADA2 MAN2B1 ARHGAP9 GMFG **LMO2** PLAC8 PTPRN2 ANPEP ARHGAP45 CFHR1

Enrichment FDR	# genes	functional category	27
0.03862997990	13	Cellular developmental process	
0.03914088927	11	Immune system process	

MIF HIST1H4I NGFR MLH1 ARHGAP4 **EGR1 BEX1** EDARADD NME2 **CD44** FERMT3 (KMT2A) S100A4

Enrichment FDR	# genes	functional category	392
0.001560480373	116	Cellular developmental process	
0.003968887858	96	Animal organ development	
0.000130929199	83	Anatomical structure morphogenesis	
0.003991861232	75	Regulation of developmental process	
0.003991861232	69	Nervous system development	
0.000130929199	42	Embryo development	

CASP8 RARB PAK3 PCK2 RND2 GDF1 RNF165 GDF7 CDH12 TNIK DDR2 **SOX7 FOXB1** BRSK2 **FOXE1** CAV3 MAPT SYT17 SFRP1 **HOXA5** APOE SERPINF1 TGFB1I1 ZNF503 INSM1 L3MBTL1 ALKAL1 COL5A2 MAGEA2 CYP26B1 SARM1 SPATA20 CELSR3 MGST1 SH2D2A HSPA5 **HIPK2** SDK2 TRIB2 CRMP1 ITGA8 ADCYAP1R1 LRP2 GNAO1 DMC1 TRIB3 RASSF2 SMARCA1 ZNF423 **FOXF1** MYH14 GSDME **CXCL12** SEPT4 CSPG5 PCSK4 **KLF7** B4GALT6 **ALDH6A1 HOXB8 HOXB5** NRN1 SEPT6 FLRT3 AMOT CCDC136 **ALDH1A2** RAMP2 BARX1 HOOK1 BRCA2 JDP2 **IRF8** RNF157 **PRDM16** GPC3 INA **DUSP15** CTF1 **ZIC3** VANGL2 DISC1 **KLF15** GABRB3 TMEM100 SPINT2 **SIX2** PAQR8 SH3PXD2B **HOXC10** DHTKD1 AMIGO1 CREB3L2 SLC4A5 TXNRD3 SMOC1 **RORB** HLA-DOA CFAP44 LAMA3 MYEF2 ITGB4 **EPCAM** CDHR1 ARPIN LZTS3 NDRG4 FZD10 ESRRB MDGA2 **KLF6** NRXN3 DSG2 PRKCB KRTAP19-1 HIST1H3E

Enrichment FDR	# genes	functional category	10
0.000683915291	4	Response to endoplasmic reticulum	

PDIA4 DDIT3 HERPUD1 DERL3

Figure S4

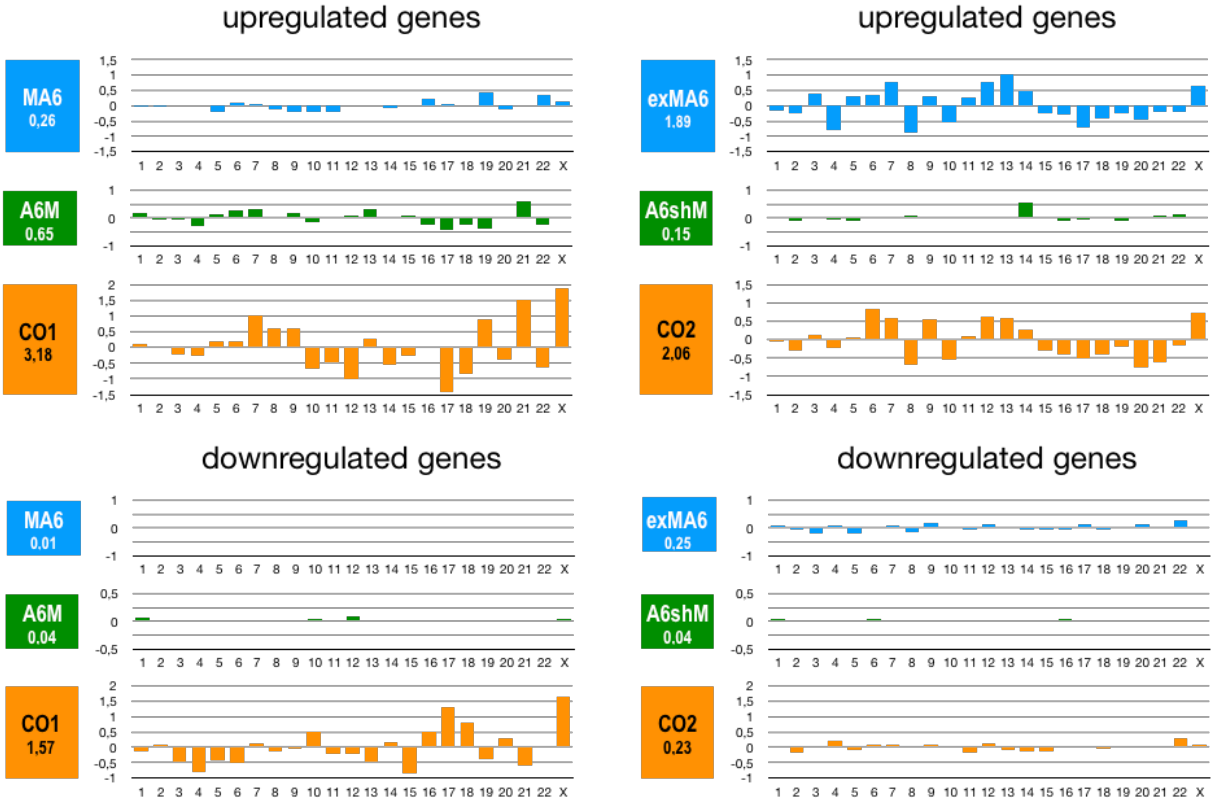

Figure S5

ATAC-Seq data dissection

	gene entries	total up	total down	log2 > 1	log2 > 2	log2 < -2	log2< -1
MLL-AF6	52,236	31,846	20,39	844	314	16	72
AF6-MLL	51,348	27,875	23,472	258	124	44	137
CO1	51,625	29,188	22,437	1	318	146	720
exMLL-AF6	51,785	28,906	22,879	276	97	35	113
AF6-shMLL	50,933	25,675	25,258	302	74	78	187
CO2	51,113	26,55	24,563	272	132	62	151

	gene entries	PG	NA genes	LINC	MIR	SNO	MT	PrCodG
MLL-AF6	52,236	9,78	17,211	1,839	1,192	241	37	21,955
AF6-MLL	51,348	9,463	16,796	1,82	1,122	219	37	21,904
CO1	51,625	9,519	16,968	1,819	1,131	238	37	21,93
exMLL-AF6	51,785	9,607	17,028	1,817	1,152	233	37	21,937
AF6-shMLL	50,933	9,312	16,623	1,818	1,077	215	37	21,867
CO2	51,113	9,357	16,729	1,815	1,066	222	37	21,901

Figure S6

MACE

	PG/NA	PCG
MA6	42	146
A6M	565	72
CO1	1,192	868
exMA6	816	404
A6shM	277	27
CO2	841	525
MA6	12	18
A6M	46	78
CO1	253	1,568
exMA6	260	354
A6shM	15	31
CO2	248	292

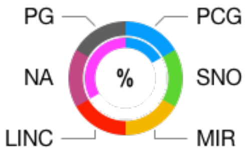

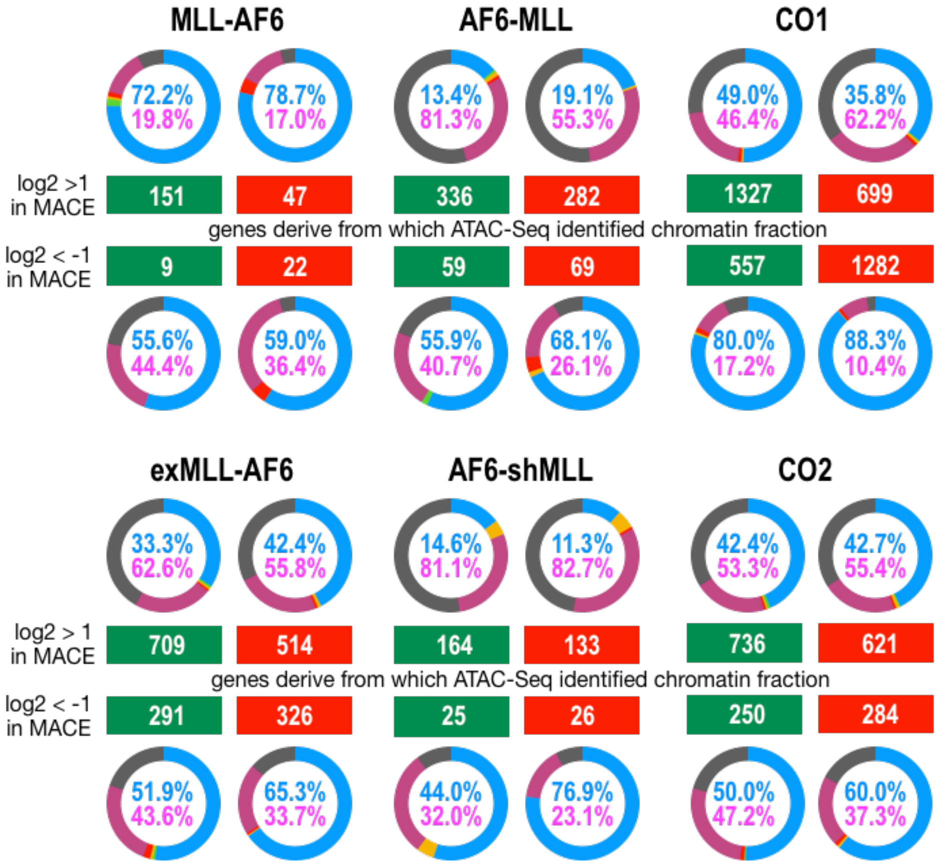

Supplement: Supplementary file 1 — Supplemental Data file [file 41388_2021_1983_MOESM1_ESM.pdf]
